# Toxicity of Certain Widely Used Antiseptics

**Published:** 1918-12

**Authors:** 


**Toxicity of Certain Widely Used Antiseptics.** By Herbert D. Taylor, M. D., and J. Harold Austin, M. D. *Journal of Experimental Medicine*, May 1, 1918.

The authors have investigated the toxic power of a certain number of usual antiseptics under conditions allowing a rapid absorption of them. Experiments were carried out on mice and guinea pigs. Injections were practised intraperitoneally with increasing doses of usual antiseptics chloramine-T, dichloramine-T, Dakin's hypochlorite, iodine, phenol. Control injections of four of the vehicles generally used — water, normal salt solution, sterile oil paraffin, Dakin's and Durham's chlorcosane, — had no bad effects, even if injected in great quantities. On the contrary, eucalyptol, formerly used as a vehicle for dichloramine-T, has a very high toxic rate and must be severely proscribed.

On the whole, the toxicity of the antiseptics follows the same order both in mice and guinea pigs. Arranged in the order of decreasing toxicity, the substances tested were : eucalyptol and brilliant green; mercurophen, mercuric chloride and chloramine-T; dichloramine-T and proflavine; hypochlorite, Javelle water; iodine and phenol.

The fatal doses of these substances having been determined experimentally for mice, and these results being extended to man, the following quantities would prove to be fatal for a man weighing 70 kg.

0.14 cc. of equal parts of paraffin oil and eucalyptol.

144 cc. of a 2 per cent. sol. of chloramine-T.

160 cc. of a 5 per cent. solution of dichloramine-T in chlorcosane.

1600 cc. of any of the hypochlorite sol. tested, having sodium hypochlorite titration of 0.5 per cent.

It must be kept in mind that the absorption by surface wounds is always very faint, and therefore no ill effects from the use of great quantities of antiseptics in external dressings need to be feared. But preceding results must be taken into account when antiseptics are used in closed cavities of the body, or sutured with a wound.

